# Awareness and utilization of genetic testing for hereditary cancers in cancer survivors: a cross-sectional 2021 HINTS-SEER study

**DOI:** 10.1007/s11764-025-01823-3

**Published:** 2025-05-22

**Authors:** Kirithiga Ramalingam, Stephen Li, Meg McKinley, Robin C. Vanderpool, Sarah H. Nash, Salma Shariff-Marco, Aaron Scheffler, Erin L. Van Blarigan, Mindy C. DeRouen

**Affiliations:** 1 Department of Surgery, University of California San Francisco, San Francisco, CA, USA; 2 Department of Epidemiology and Biostatistics, University of California San Francisco, San Francisco, CA, USA; 3 Greater Bay Area Cancer Registry, University of California San Francisco, 550 16. Street, San Francisco, CA #263694158, USA; 4 Health Communication and Informatics Research Branch, Division of Cancer Control and Population Sciences, Behavioral Research Program, National Cancer Institute, Rockville, MD, USA; 5 Department of Epidemiology, College of Public Health, University of Iowa, Iowa City, IA, USA

**Keywords:** Cancer genetic testing, Sociodemographic factors, HINTS-SEER, Cancer survivors

## Abstract

**Purpose:**

We aimed to identify factors associated with genetic testing awareness and use among a large sample of US cancer survivors participating in NCI’s Health Information National Trends Survey of cancer survivors identified through the Surveillance, Epidemiology, and End Results program, a unique pilot study expanding the number of cancer survivors typically captured by HINTS.

**Methods:**

We analyzed 2021 HINTS-SEER data to determine sociodemographic factors associated with awareness and utilization of germline genetic testing using survey-weight-adjusted multivariable logistic regression.

**Results:**

Of 1232 survivors (any site), the majority had breast (23%) or prostate (23%) cancer. Among the overall study population, 77% were aware of and 24% utilized genetic testing. Females and those with a college education, income over $100,000, or family history of cancer had greater odds of awareness compared to males, those with less than college education, income under $20,000, or no family history, respectively. Among prostate cancer survivors, those ≥ 65 years in age had lower odds of awareness. Females and the privately insured had greater odds of utilization compared to males and publicly insured, respectively. Those ≥ 65 years or retired had lower odds of utilization compared to < 65 years and being employed, respectively.

**Conclusion:**

We identified sociodemographic factors associated with awareness and utilization of germline genetic testing among cancer survivors. Findings warrant further investigation to understand mechanisms underlying disparities in awareness and use of genetic testing.

**Implications for Cancer Survivors:**

This study highlights cancer survivor populations that may require additional support around awareness of and use of germline genetic testing

## Introduction

Hereditary cancers, those attributable to genetic mutations passed through families, account for 5–10% of new cancer diagnoses [1]. Germline genetic testing, performed through a blood or saliva sample, offers a powerful tool for identifying individuals at an increased risk for hereditary cancers as well as informing treatment decisions among those with a cancer diagnosis [2]. Studies have shown genetic testing can improve survival rates in patients diagnosed with cancer by guiding decisions on prophylactic surgeries such as bilateral mastectomy and oophorectomy and enabling personalized treatment with targeted cancer therapies for BRCA1/2 mutation carriers [3]. Additionally, testing allows for early identification of cancer risk among relatives of patients diagnosed with hereditary cancers. This information can be used to tailor screening recommendations and potentially prevent cancer altogether [4–7].

National guidelines, such as those from the National Comprehensive Cancer Network (NCCN), recommend germline testing for all patients diagnosed with male breast cancer, ovarian cancer, and pancreatic cancer [8]. Testing is also recommended for select groups with breast (e.g., strong family history of breast), prostate (metastatic), and colorectal (e.g., early onset) cancers [8, 9]. The American Society of Breast Surgeons advocates for genetic testing in all breast cancer patients [10]. However, research reveals a low prevalence of testing among cancer patients in the United States (U.S.), with an overall prevalence of only 7% [11, 12]. While the decision to undergo genetic testing is guided by personal preference and values, some factors that prior research has shown to be associated with genetic testing for cancer suggest alternate influences and information needs that may hinder informed decision making: age, gender, race and ethnicity, marital status, family history of cancer, education, income, occupation, and insurance status have all been shown to be associated with germline genetic testing for cancer [12–16]. To improve access to and utilization of germline genetic testing, analyzing a recent survey among cancer patients can offer valuable insights into the factors influencing cancer patients’ awareness and utilization of this potentially lifesaving tool.

## Methods

### HINTS-SEER database/study population

In this study, we analyzed data from the Health Information National Trends Survey (HINTS)-Surveillance, Epidemiology, and End Results (SEER) project conducted by the National Cancer Institute (NCI) in 2021 [17]. The HINTS-SEER dataset was specifically tailored to include cancer survivors diagnosed with any cancer before 2018 and administered the HINTS 5 Cycle 4 survey plus additional questions specific to cancer survivorship. Participants were recruited from three SEER registries to construct this dataset: Iowa, New Mexico, and the Greater Bay Area. The NCI strategically selected these regions to ensure a diverse representation of race, ethnicity, and geographic characteristics among the study population [18]. To be eligible, individuals had to be adults alive at the time of recruitment as of December 2020, diagnosed with invasive cancer (excluding non-melanoma skin cancer), and have a date of last contact by the registry later than January 1, 2016. Data collection occurred between January 11, 2021, and August 20, 2021; the overall HINTS-SEER survey response was 12.6%. To ensure the generalizability of findings to the population of female breast cancer patients, two survey responses from individuals reporting breast cancer and male gender by birth were excluded. The present objective was to investigate the sociodemographic factors associated with awareness and utilization of germline genetic testing among cancer survivors.

### Variables

The outcome variables were awareness and utilization of germline genetic testing. The survey question to address awareness of germline genetic testing was “Genes are inherited from your parents and are passed down from one generation to the next. Genetic tests can determine your genetic makeup. Have you heard of cancer genetic testing (for example, testing for inherited cancer syndromes like BRCA1/2 or Lynch Syndrome)?” The survey question to address use of germline genetic testing was “Have you had cancer genetic testing (for example, testing for inherited cancer syndromes like BRCA1/2 or Lynch Syndrome)?” For each question, participants were asked to mark all that applied from the following options: “(1) Ancestry testing: To determine the background or geographic/ethnic origin of an individual’s ancestors (for example, Ancestry.com and 23andMe); (2) Genetic health risk testing: To determine health risk for a variety of health conditions (for example, 23andMe); (3) Cancer genetic testing: (for example, testing for inherited cancer syndromes like BRCA1/2 or Lynch Syndrome); (4) Other – Specify; (5) Not sure; (6) I have not heard of any of these types.” The full survey is available online: “HINTS-SEER (2021) Full-content, English Version” at https://hints.cancer.gov/data/survey-instruments.aspx#H2021_SM.

Predictor variables considered included age, gender assigned at birth, education, occupation, insurance, income, marital status, and family history of cancer. Participants were categorized into three age groups: < 40 years, 40–64 years, and 65 years and older. We aggregated response options to survey questions related to education, occupation, insurance, and marital status to account for small cell counts. Educational attainment was dichotomized as less than a college degree and a college degree or higher. Occupational status was classified into three categories: employed, retired, and other (unemployed, student, homemaker, disabled, and other). Health insurance status was categorized as uninsured, publicly insured, and privately insured. Marital status was dichotomized as married and other (single, divorced, widowed, separated, living as married, or living with a romantic partner). The category values of “living as married” or “living with a romantic partner” together constituted only 3% of the total responders. Family history was defined as a positive history of cancer in any of the first- or second-degree biological relatives (parents, brothers and sisters, children, grandparents, aunts and uncles, nieces, and nephews).

### Statistical analyses

Using survey-weighted data, we calculated awareness and utilization of germline genetic testing in the overall cancer survivor cohort and by specific cancer types. In the HINTS-SEER study, weighting was applied to participants based on control totals from eligible populations within each of the three registries. These weights reflect the specific registry population rather than the overall state population. When comparing HINTS-SEER estimates to other HINTS administrations, caution is advised due to differences in weighting methods. Cancer survivors who completed HINTS-SEER questionnaires received full-sample weights and 50 replicate weights. The full-sample weight is used for population and subpopulation estimates, while replicate weights help compute standard errors. Sampling weights ensure valid inferences from the sample to the eligible population, accounting for nonresponse and noncoverage biases. The computation of full-sample weights involves base weight calculation, nonresponse adjustment, and calibration to registry-specific control totals. Replicate weights are derived using the “delete one” jackknife method. Further details of the weighting process can be found on the HINTS-SEER (2021) methodology report PDF on the webpage https://hints.cancer.gov/data/survey-instruments.aspx#H2021_SM.

We assessed the association of various socio-demographic factors (predictors) with awareness and utilization outcomes. To achieve this, we employed multivariable logistic regression models that incorporated jackknife replication weights for robust estimation [17]. For each predictor, a unique model was constructed, adjusting for relevant covariates based on minimal sufficient adjustment sets derived from a Directed Acyclic Graph (DAG) informed by prior research and expert insights [12–14, 19–27]. This approach ensures robust analyses by including only the essential confounding variables and resulted in the following models: Models assessing gender were adjusted for survey weights. Models assessing age at diagnosis were adjusted for survey weights and family history. Models assessing education were adjusted for survey weights, age, gender, and race. Models assessing occupation were adjusted for survey weights, age, gender, education, and race. Models assessing insurance status were adjusted for survey weights, age, education, gender, marital status, occupation, and income. Models assessing income were adjusted for survey weights, education, and occupation. Models assessing marital status were adjusted for survey weights, age, education, gender, insurance, occupation, and income. Models assessing family history were adjusted for survey weights.

Recognizing unique guidelines and considerations for genetic testing in different cancers, we performed similar analyses on breast and prostate cancer survivors as they had sufficient number of outcomes [8, 28]. By stratifying our analyses, we aimed to identify any unique sociodemographic patterns or barriers to genetic testing specific to these cancer survivor sub-populations. We performed multivariable-adjusted analyses for breast cancer survivors to explore factors influencing both awareness and utilization. For prostate cancer survivors, due to the limited number of outcomes for utilization of germline genetic testing, we only analyzed the outcome on awareness. We could not analyze the relationship between race and ethnicity and our outcome variables due to the small size of some groups. We were also unable to examine the association between factors such as income, marital status, family history, and the outcome of awareness of germline genetic testing among survivors of prostate cancer.

## Results

**Fig. 1:**
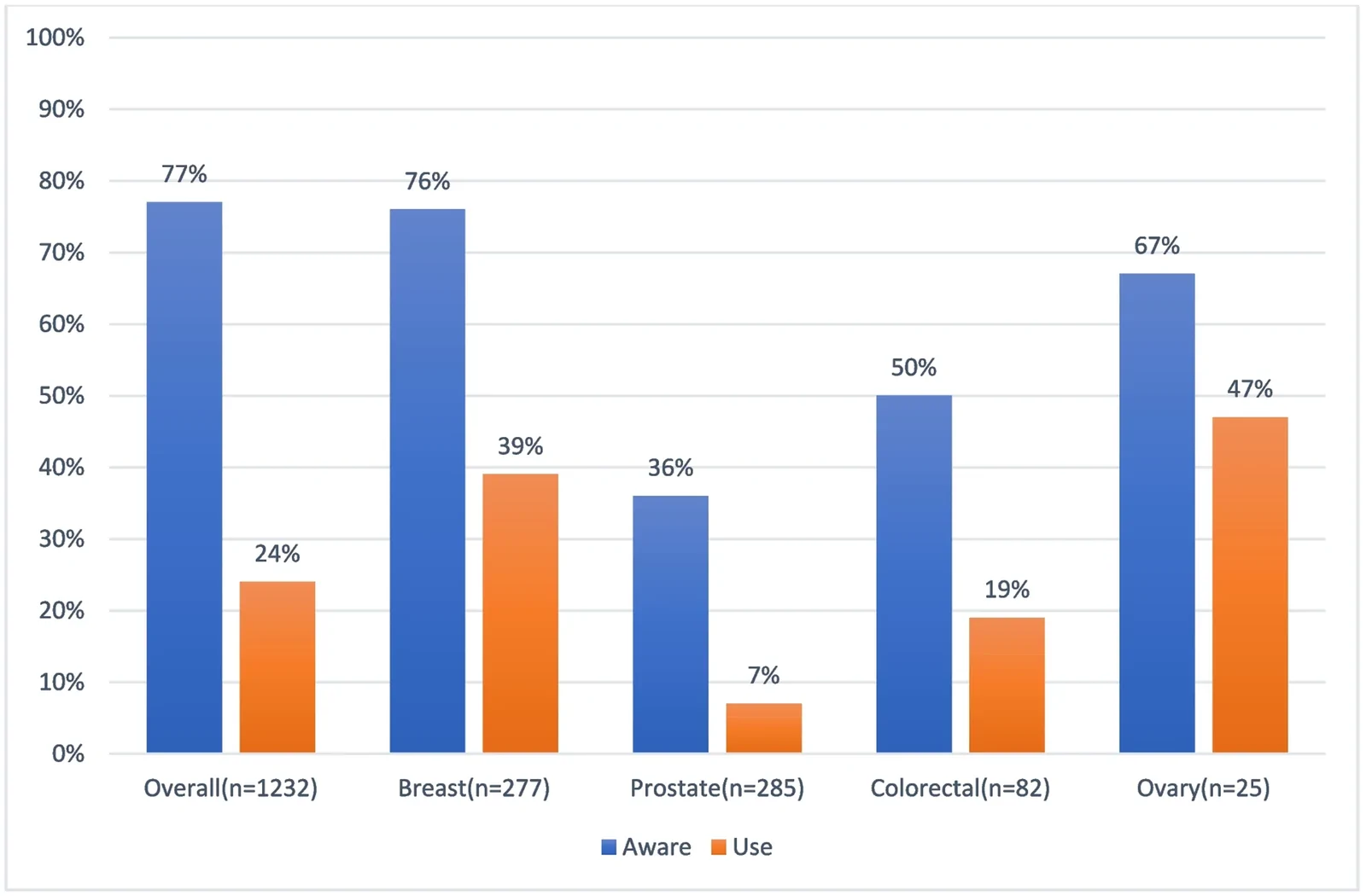
Awareness and use of germline genetic testing among cancer survivors: HINTS-SEER study, HINTS Health Information National Trends Survey; SEER, Surveillance, Epidemiology, and End Results; %, weighted percentages

A total of 1232 cancer survivors responded to the survey. Among these, 23% (*n* = 277) reported a history of breast cancer, 23% (*n* = 285) of prostate cancer, 6.7% (*n* = 82) of colorectal cancer, and 2% (*n* = 25) of ovarian cancer (Table 1). Other cancer types among survivors represented in the “Overall” category included uterine/endometrial (*n* = 68), lymphoma (*n* = 63), thyroid (*n* = 45), kidney (*n* = 43), head and neck (*n* = 38), leukemia (*n* = 36), and lung (*n* = 28); Supplementary Table 1 shows all cancer types reported and their frequency in the study population. A majority of participants were female gender (54%), aged 40–64 years (56%), non-Hispanic White (70%), with a college degree or higher (77%), retired (58%), and had public insurance (66%). A high proportion (80%) reported a family history of cancer. Although 77% of survivors were aware of genetic testing for hereditary cancers, only 23.5% had used it (Fig. 1). The highest awareness was seen among breast (76%) and ovarian (67%) cancer survivors, who also reported greater use (39% and 47%, respectively).

With multivariable analysis of the overall cohort, female gender (OR = 3.1; 95% CI 2.3–4.3), college or higher education (OR = 2.5; 95% CI 1.6–3.9), annual income greater than $100,000 (OR = 3.3, 95% CI 1.9–5.9), and family history of cancer (OR = 2.0; 95% CI 1.4–3.0) were significantly associated with greater odds of awareness of genetic testing compared to male gender, less than college education, annual income less than $20,000, absent or unsure family history of cancer respectively (Table 2). Conversely, age ≥ 65 years, particularly among prostate cancer survivors, was associated with lower odds of awareness compared to age < 65 years (OR = 0.4; 95% CI 0.4–0.8). Overall, female gender (OR = 5.1; 95% CI 3.2–8.2) and private insurance (OR = 1.6; 95% CI 1.1–2.5) were associated with greater odds of testing utilization compared to male gender and public insurance, respectively (Table 3). Age ≥ 65 years (vs. age < 40 years, OR = 0.4; 95% CI 0.2–0.7) and retired status (OR = 0.4; 95% CI 0.3–0.6) were associated with lower odds of utilization compared to age < 65 years and being employed. In contrast, age ≥ 65 years (OR = 0.4; 95% CI 0.2–0.7) and being retired (OR = 0.4; 95% CI 0.3–0.6) were associated with lower odds of utilization compared to age < 65 years and employed status. Similar trends were observed among breast cancer survivors specifically, with private insurance (vs. public insurance, OR = 2.0, 95% CI: 1.0–3.9) associated with greater odds of utilization and age ≥ 65 years (vs. age < 40 years, OR = 0.3, 95% CI 0.1–0.8) associated with lower odds of utilization. Marital status was not associated with awareness or use of genetic testing.

## Discussion

This study used data from the HINTS-SEER project to examine the sociodemographic factors associated with awareness and utilization of germline genetic testing among cancer survivors. Our study is unique in that it focuses exclusively on cancer survivors, and the results represent three major areas from which the samples were drawn, namely Iowa, New Mexico, and the Greater Bay Area. Our findings align with previous research, highlighting disparities in patient access and knowledge.

Similar to prior studies, men were less likely to be aware of and receive genetic testing compared to women [12, 13, 15]. This may be due to a historical focus on promoting genetic testing in women, particularly for breast and ovarian cancers [29]. We observed lower awareness and testing among older survivors, a finding common among other studies [13, 15, 16, 30]. This could be due to several factors. Older adults may have lower exposure to recent medical information about genetic testing compared to younger individuals [31]. Additionally, mobility limitations and lack of adequate insurance may hinder access to healthcare facilities where testing is offered [32]. Notably, a lower threshold for ordering genetic testing often exists for younger patients due to the earlier onset of hereditary cancers in this age group [33].

Lower education and income were associated with decreased awareness of genetic testing similar to the findings seen in other studies [13, 15, 16]. This highlights potential disparities in access to genetic counseling and educational resources, crucial for informed decision-making about genetic testing. Our study found no significant association between marital status and genetic testing after adjusting for other factors. A sensitivity analysis categorizing the small number of respondents reporting “Living as married” or “Living with a romantic partner” as “Married” did not change this observation. This results contradict a previous study, suggesting that marital status may not be a primary predictor but might influence testing through other variables that we adjusted for—namely age, education, gender, insurance, occupation, and household income [13].

The observed prevalence of testing among female breast, colorectal, and ovarian cancer survivors was comparable to a recent large-scale study encompassing nearly one million cancer patients from California and Georgia [12]. This consistency strengthens the validity of our findings and suggests a general trend in genetic testing uptake within this population segment. Despite increasing awareness of germline genetic testing over time, a significant disparity persists between those who are aware and those who actually undergo testing [13, 15, 34, 35]. In our study, focusing on breast and ovarian cancers where germline genetic testing is recommended for select groups, we identified a disparity between the proportion of survivors who were aware of the testing (76% for breast and 67% for ovary) and the proportion of those who received it (39% for breast and 47% for ovary). This gap highlights additional barriers beyond simple awareness that impede utilization. Our analysis identified insurance status as one such significant barrier. After adjusting for factors like education, occupation, and income, we found a clear association between having private insurance, compared to public insurance, and greater odds of having undergone genetic testing. This aligns with findings from the National Health Interview Survey, which demonstrated a positive correlation between private insurance and increased testing rates [14]. Similar disparities based on insurance type have been reported in studies focused on ovarian cancer [36, 37]. These findings suggest that insurance coverage is critical in facilitating access to genetic testing. Individuals with public insurance may face financial limitations that hinder their ability to pursue testing, even if they know its potential benefits.

Results should be considered in light of strengths and limitations. The complex survey design and sampling weights within the HINTS-SEER project enhance the generalizability of our findings to cancer survivors residing in the three geographic regions included. Stratifying the analyses by cancer type allowed us to explore potential variations in awareness and utilization patterns among survivors of breast and prostate cancers with established genetic testing guidelines. However, although the survey response rate is similar to prior studies, selection bias remains a potential concern. Additionally, the focus on three registries, while they represent distinct segments of the U.S. population, limits the generalizability of the study findings to the entire U.S. population. Since most patients undergo germline genetic testing around the time of initial diagnosis of cancer, there is a potential for measurement error of self-reported data, particularly given the time from diagnosis to study enrollment. The study’s cross-sectional nature also presents a limitation by preventing causal inference. Furthermore, due to the relatively small sample sizes within specific categories, such as race and ethnicity, the study could not determine relationships between these factors and awareness/utilization of genetic testing. Due to lack of survey questions capturing indications for germline genetic testing such as personal and family history of certain cancer subtypes, microsatellite instability tumors, or presence of polyps in the HINTS-SEER database, we could not exclusively include survivors eligible for germline genetic testing under current clinical guidelines in this study. Finally, it is essential to acknowledge that our study did not address other barriers to genetic testing previously reported in the literature, such as patient anxiety, healthcare mistrust, or genetic information misuse, nor did it address provider knowledge or counselor availability. Future research should investigate these factors for a more complete picture [38–40].

## Conclusion

This study highlights the sociodemographic factors associated with genetic testing awareness and utilization among a large sample of U.S. cancer survivors. By acknowledging the limitations and incorporating insights from this study, future research can build upon these findings to further guide targeted interventions to improve the utilization of germline genetic testing for inherited cancers.

## Supplementary Material

Supplementary Table 1

The online version contains supplementary material available at https://doi.org/10.1007/s11764-025-01823-3.

## Figures and Tables

**Table 1:** Sociodemographic characteristics of 1232 cancer survivors with weighted percentages, overall and by cancer site

	*n* (Wgt %)				
Demographic/clinical characteristics	Overall 1232 (100%)	Breast 277 (23%)	Prostate 285 (23%)	Colorectal 82 (7%)	Ovary 25 (2%)
Gender^a^					
Male	568 (45)	- †	285 (100)	40 (47)	-
Female	645 (54)	277 (100)	-	42 (53)	25 (100)
Age at cancer diagnosis^a^					
< 40 years old	132 (14)	29 (15)	* (2)	*	*
40–64 years old	706 (56)	184 (65)	147 (49)	52 (65)	18 (69)
65 and above	347 (26)	58 (19)	115 (42)	25 (26)	*
Race/ethnicity^a^					
Non-Hispanic White	905 (70)	197 (70)	212 (70)	51 (59)	17 (54)
Hispanic	137 (11)	41 (13)	26 (10)	9 (6)	*
Non-Hispanic Asian	64 (7)	13 (6)	13 (6)	13 (19)	*
Non-Hispanic Black	15 (3)	*	* (5)	*	*
Non-Hispanic another race	13 (1)	*	*	*	*
Education^a^					
Less than College	264 (20)	65 (22)	44 (14)	23 (27)	*
College or Greater	936 (77)	209 (76)	230 (81)	59 (73)	20 (86)
Occupation^a^					
Employed	295 (28)	70 (30)	45 (17)	13 (18)	11 (57)
Retired	770 (58)	178 (61)	212 (71)	57 (64)	* (28)
Others^b^	122 (11)	24 (7)	11 (6)	* (16)	*
Insurance^a^					
Uninsured	* (1)	*	*	*	*
Public Insurance	805 (66)	176 (65)	174 (61)	597(74)	17 (67)
Private insurance	362 (28)	87 (30)	96 (33)	21 (23)	* (21)
Income^a^					
Less than $20,000	102 (7)	24 (8)	17 (5)	*	*
$20,000 to $49,999	274 (21)	69 (23)	48 (17)	24 (32)	*
$50,000 to $99,999	401 (32)	94 (33)	94 (31)	19 (24)	11 (36)
$100,000 or more	444 (39)	86 (34)	122 (46)	33 (38)	* (42)
Marital Status^a^					
Married	792 (64)	155 (56)	207 (72)	61 (76)	18 (70)
Others^c^	402 (33)	119 (42)	64 (23)	21 (24)	* (30)
Family History^a^					
Yes	1000 (80)	240 (84)	220 (75)	62 (71)	20 (81)
No/unsure	218 (19)	35 (15)	61 (23)	19 (28)	*

*Wgt* survey weight adjusted

a Number of participants with missing responses: gender assigned at birth < 11, age = 47, race = 98, education = 32, occupation = 45, insurance = 55, income = 11, marital status = 38, family history = 14

b Unemployed, homemaker, student, disabled, other

c Divorced, widowed, separated, single, living with a romantic partner

† For simplicity, we omitted male breast cancer survivors from the analysis (*n* <11)

* Cell count ≤ 10

**Table 2:** Factors associated with awareness of germline genetic testing

Sociodemographic/clinical characteristics	Overall		Breast		Prostate	
	Aware *N* (Wgt %)	Adjusted odds ratio^a^ (95% CI)	Aware *N* (Wgt %)	Adjusted odds ratio^a^ (95% CI)	Aware *N* (Wgt %)	Adjusted odds ratio^a^ (95% CI)
Gender^a^						
Male	224 (18)	Ref	-	NA	110 (36)	NA
Female	435 (36)	3.14 (2.31, 4.26)	213 (76)	NA	-	NA
Age at cancer diagnosis^b^						
< 40 years old	84 (9)	Ref	173 (63)	Ref	77 (25)	Ref
40–64 years old	433 (34)	0.83 (0.54, 1.29)				
65 and above	139 (10)	0.36 (0.21, 0.61)	39 (13)	0.52 (0.25, 1.08)	32 (11)	0.40 (0.21, 0.78)
Education^c^						
Less than college	96 (7)	Ref	41 (14)	Ref	12 (4)	Ref
College or greater	562 (46)	2.50 (1.63, 3.85)	172 (62)	2.28 (0.82, 6.34)	96 (31)	1.72 (0.65, 4.53)
Occupation^d^						
Employed	197 (19)	Ref	64 (27)	Ref	19 (7)	Ref
Retired	393 (29)	0.67 (0.44, 1.02)	128 (42)	0.25 (0.05, 1.23)	82 (24)	1.10 (0.52, 2.33)
Others ^i^	63 (6)	0.72 (0.31, 1.65)	19 (6)	1.22(0.18, 8.29)	*	2.14 (0.16, 27.98)
Insurance^e^						
Public Insurance	141 (51)	Ref	141 (51)	Ref	70 (23)	Ref
Private insurance	68 (23)	1.13 (0.90, 1.43)	68 (23)	1.02 (0.43, 2.43)	38 (12)	1.21 (0.60, 2.44)
Income^f^						
Less than $20,000	10 (4)	Ref	10 (4)	Ref	*	
$20,000 to $49,999	52 (17)	2.09 (1.15, 3.79)	52 (17)	3.37 (0.77, 14.68)	13 (3)	NA
$50,000 to $99,999	75 (26)	2.43 (1.43, 4.14)	75 (26)	3.96 (0.95, 16.59)	35 (11)	NA
$100,000 or more	76 (29)	3.29 (1.85, 5.85)	76 (29)	4.64 (0.87, 24.64)	55 (19)	NA
Marital status^g^						
Married	451 (36)	1.18 (0.82, 1.71)	131 (47)	2.36 (0.83, 6.70)	*	NA
Others^j^	200 (16)	Ref	80 (28)	Ref	*	NA
Family history^h^						
Yes	577 (47)	2.00 (1.36, 2.95)	577 (47)	0.82 (0.31, 2.16)	*	NA
No/unsure	86 (8)	Ref	86 (8)	Ref	*	NA

*Wgt* survey weight adjusted

a Model adjusted for survey weights

b Model adjusted for survey weights and family history

c Model adjusted for survey weights, age, gender, and race

d Model adjusted for survey weights, age, gender, education, and race

e Model adjusted for survey weights, age, education, gender, marital status, occupation, and income

f Model adjusted for survey weights, education and occupation

g Model adjusted for survey weights, age, education, gender, insurance, occupation, and income

h Model adjusted for survey weights, none

i Other category includes unemployed, homemaker, student, disabled, other

j Other category includes divorced, widowed, separated, single, living as married, living with a romantic partner

* Cell count ≤ 10

**Table 3:** Factors associated with use of germline genetic testing

Sociodemographic/clinical characteristics	Overall		Breast	
	Use *N* (Wgt %)	Adjusted odd ratio^a^ (95% CI)	Use *N* (Wgt %)	Adjusted odd ratio^a^ (95% CI)
Gender^a^				
Male	31 (3)	Ref	-	NA
Female	171 (14)	5.13 (3.19, 8.24)	112 (39)	NA
Age at cancer diagnosis^b^				
< 40 years old	32 (3)	Ref	100 (35)	Ref
40–64 years old	141 (11)	0.84 (0.50, 1.42)		
65 and above	30 (2)	0.38 (0.21, 0.69)	11 (3)	0.30 (0.11, 0.82)
Education^c^				
Less than College	28 (3)	Ref	20 (8)	Ref
College or Greater	174 (14)	1.02 (0.57, 1.84)	92 (31)	1.22 (0.53, 2.80)
Occupation^d^				
Employed	83 (8)	Ref	44 (19)	Ref
Retired	98 (7)	0.37 (0.25, 0.56)	58 (18)	0.28 (0.14, 0.55)
Others ^i^	21 (2)	0.53 (0.27, 1.03)	* (3)	0.43 (0.11, 1.73)
Insurance^e^				
Public Insurance	138 (12)	Ref	74 (26)	Ref
Private insurance	63 (5)	1.61 (1.06, 2.45)	37 (13)	2.02 (1.03, 3.93)
Income^f^				
Less than $20,000	13 (1)	Ref	* (2)	Ref
$20,000 to $49,999	41 (3)	0.82 (0.31, 2.16)	28 (8)	0.55 (0.09, 3.33)
$50,000 to $99,999	64 (5)	0.78 (0.27, 2.24)	35 (12)	0.48 (0.09, 2.70)
$100,000 or more	87 (8)	0.87 (0.33, 2.32)	41 (17)	0.76 (0.14, 4.21)
Marital status^g^				
Married	131 (12)	1.27 (0.82, 1.97)	69 (26)	1.70 (0.88, 3.28)
Others^j^	68 (5)	Ref	42 (12)	Ref
Family history^h^				
Yes	179 (14)	1.21 (0.63, 2.33)	99 (32)	0.68 (0.22, 2.13)
No/unsure	25 (3)	Ref	13 (7)	Ref

*Wgt* survey weight adjusted

a Model adjusted for survey weights

b Model adjusted for survey weights and family history

c Model adjusted for survey weights, age, gender, and race

d Model adjusted for survey weights, age, gender, education, and race

e Model adjusted for survey weights, age, education, gender, marital status, occupation, and income

f Model adjusted for survey weights, education and occupation

g Model adjusted for survey weights, age, education, gender, insurance, occupation, and income

h Model adjusted for survey weights, none

i Other category includes unemployed, homemaker, student, disabled, other

* Cell count ≤ 10

## Data Availability

The HINTS-SEER instrument, supporting documentation, and full methodology report are available for public use at http://hints.cancer.gov.
