# Supplementary material for: Awareness and utilization of genetic testing for hereditary cancers in cancer survivors: a cross-sectional 2021 HINTS-SEER study
Source: J Cancer Surviv. 2025 May 22;20(Suppl 1):357–65. doi: 10.1007/s11764-025-01823-3 (PMC12754848; doi:10.1007/s11764-025-01823-3)
Supplement: Supplementary file 1 [file 11764_2025_1823_MOESM1_ESM.docx]

**Supplementary Table 1: Types of cancers and number of survivors in study population.**

| **Cancer type** | **N** |
| --- | --- |
| Breast | 277 |
| Prostate | 285 |
| Colorectal | 82 |
| Ovary | 25 |
| Head and neck | 38 |
| Esophagus, stomach and small intestine | <11 |
| Anus | 12 |
| Exocrine pancreas | <11 |
| Liver | <11 |
| Lung | 28 |
| Cutaneous | 126 |
| Melanoma | <11 |
| Soft tissue | <11 |
| Vulva | 28 |
| Cervix | <11 |
| Uterine/endometrial | 68 |
| Fallopian tube | <11 |
| Testis | 17 |
| Kidney | 42 |
| Bladder | 25 |
| Thyroid | 45 |
| Lymphoma | 63 |
| Leukemia | 36 |
| Others* | 27 |

*includes thymus, bone, peritoneal, nipple, other adnexa, penis, ureter, eye, brain, plasmacytoma, myeloma, carcinoma of unknown origin.
